# Some Evidence for an Association Between Early Life Adversity and Decision Urgency

**DOI:** 10.3389/fpsyg.2019.00243

**Published:** 2019-02-11

**Authors:** Johanne P. Knowles, Nathan J. Evans, Darren Burke

**Affiliations:** ^1^School of Psychology, University of Newcastle, Callaghan, NSW, Australia; ^2^Department of Psychology, University of Amsterdam, Amsterdam, Netherlands

**Keywords:** early life adversity, decision urgency, life history theory, cognitive modeling, evidence accumulation models

## Abstract

The relationship between early life adversity and adult outcomes is traditionally investigated relative to risk and protective factors (e.g., resilience, cognitive appraisal), and poor self-control or decision-making. However, life history theory suggests this relationship may be adaptive—underpinned by mechanisms that use early environmental cues to alter the developmental trajectory toward more short-term strategies. These short-term strategies have some theoretical overlap with the most common process models of decision-making—evidence accumulation models—which model decision urgency as a decision threshold. The current study examined the relationship between decision urgency (through the linear ballistic accumulator) and early life adversity. A mixture of analysis methods, including a joint model analysis designed to explicitly account for uncertainty in estimated decision urgency values, revealed weak-to-strong evidence in favor of a relationship between decision urgency and early life adversity, suggesting a possible effect of life history strategy on even the most basic decisions.

## Introduction

The association between childhood adversity and poorer physical, psychological and social outcomes across the lifespan is well-established in both research and clinical settings (see for examples Edwards et al., 2003; Koenen et al., 2010; Danese and McEwen, 2012; Centers for Disease Control and Prevention [CDC], 2018). In general, these sources of literature appear to converge on a similar conclusion: that this is a “dose-dependent” relationship where the more adverse childhood events experienced, the greater the probability, and effect, of negative outcomes (Lovallo et al., 2012; Hemmingsson et al., 2014; Centers for Disease Control and Prevention [CDC], 2018). Traditionally, these outcomes have been viewed as a consequence of a lack of self-control or poor decision-making with interventions designed around this paradigm (Mischel et al., 2011; Kidd et al., 2013).

However, an increasing body of research across a broad range of disciplines including psychology (Kidd et al., 2013; Mittal et al., 2015), biology (Fawcett et al., 2012), and economics (Anand and Lea, 2011; Bertrand et al., 2016) suggest that these “poor decision strategies” may actually reflect adaptive behavior based upon the availability of resources in the childhood environment (Griskevicius et al., 2011; Kidd et al., 2013; Mittal et al., 2015). These suggestions fall in line within a prominent evolutionary-developmental framework known as *life history theory* (Stearns, 1976; Roff, 1992; Nettle and Frankenhuis, 2019); which stems from extensive non-human biological research, and proposes that an organism’s allocation of time and energy is a consequence of a variety of environmental cues and competing goals (e.g., fitness vs. reproduction) (Stearns, 1976; Roff, 1992). While initially used to explore between species differences (Cole, 1954; Mathot and Frankenhuis, 2018), life history theory has more recently been applied to research exploring within species differences, where environmental cues in the early environment are thought to activate physiological and behavioral changes that ideally leave the individual best situated to succeed in their adult environment (Denver and Middlemis-Maher, 2010; Nettle et al., 2013).

When applied to humans, life history theory forms the overarching framework for a range of theories and empirical research aimed at understanding the relationship between the early environment and outcomes across the lifespan (Nettle et al., 2013; Bateson et al., 2014; Del Giudice, 2014). For example, psychosocial acceleration theory (Belsky et al., 1991) proposes psychosocial stress (e.g., insecure attachment, marital discord) act as cues to inadequate resources, leading to earlier puberty and earlier engagement in reproductive strategies. Proposing similar consequences, the Adaptive Calibration model, for example, implicates accelerated somatic aging (as a consequence of early life stress) (see for example, Del Giudice et al., 2011; Dańko et al., 2018). Collectively, however, while acknowledging the difficulty of disentangling the effects of genetic and epigenetic mechanisms on these outcomes, these theories (and associated research) imply that resource availability in the early environment has a profound and measurable effect on the acquisition and use of resources (e.g., food, mating opportunities) over time (see for example Belsky et al., 1991; Hill et al., 2008; Brumbach et al., 2009; Del Giudice, 2014).

More specifically, individuals with stable early environments, where resources are readily available, will be more likely to adopt cautious, long-term strategies, where short-term benefits can be sacrificed for greater long-term gain (Chisholm, 1993; Brumbach et al., 2009; Griskevicius et al., 2013). In contrast, individuals facing early life adversities, (e.g., poverty, food insecurity or physical and/or emotional abuse) appear to be more likely to adopt short-term strategies, preferring smaller rewards immediately over the possibility of greater rewards in the future (Hill et al., 1994; Chisholm, 1999; Kidd et al., 2013). Theoretically, these strategic differences are thought to be underpinned by time sensitivity or time preference (i.e., the need to consider the likelihood of immediate vs. future success of resource acquisition and to spend energy accordingly) (Chisholm, 1999). Thus, time preference is conceptualized as a key of aspect of an individual’s life history strategies (Chisholm, 1993; Hill et al., 1994; Hill and Newlin, 2002; Kruger et al., 2008). A plausible mechanism by which differences in life history strategy might manifest themselves is differences in decision urgency. Those adopting a slow life history strategy can afford to accumulate evidence until the best decision can be made, but those primed by environments of low, unpredictable resources might be better served by simply making rapid decisions based on the evidence immediately available.

Although there is some evidence linking early life adversity to more urgent high level decision strategies in later life, such as gambling (Griskevicius et al., 2011), substance use/addiction (Hill et al., 1994; Mersky et al., 2013) and impulsivity (Paál et al., 2015), no studies to date have investigated how these adversities alter the *process* of decision-making. Importantly, the last several decades of research within rapid decision-making, and decision-making more generally, have had a heavy focus on understanding the process by which humans make decisions. One of the most dominant explanations of the process of decision-making has been evidence accumulation models (EAMs), which propose that decisions are made based on the accumulation of evidence for the different alternatives from the environment. The evidence for each alternative continues to accumulate at some rate (known as the “drift rate”) until one reaches a pre-determined threshold amount of evidence (known as the “decision threshold”), resulting in a decision being triggered for that alternative.

The components of this proposed process also have important psychological meaning. The drift rate is reflective of the ease of the task for the decision-maker, with drift rates for correct alternatives being higher for easier tasks (Ratcliff, 1978) and more intelligent participants (van Ravenzwaaij et al., 2011). The decision threshold is thought to be under the control of the decision-maker, and reflects the level of urgency to make decisions. Lower thresholds reflect urgent decision strategies, with less evidence being required to trigger a decision, making them faster and less accurate, whereas higher thresholds reflect more cautious decision strategies, being slower and more accurate. This model has implications in terms of life history theory, in that, as noted earlier, individuals predisposed by conditions in their early life environment to make faster decisions, may be making those decisions based on information in the current environment information, with a lower decision threshold (less evidence) than those who are primed to make more careful decisions.

With this in mind, our study aims to investigate whether early life adversity impacts upon the very basic algorithms that underlie decision-making, or whether the impact of early life adversity found in higher-level processes is not captured within rapid decision-making. As discussed previously, research has found that early life adversity is linked to a greater focus on short-term rewards, though these studies have not assessed (1) how early life adversity impacts upon the decision process itself, or (2) whether the impact of early life adversity influences the most basic, low-level decisions. Here, we use a life history questionnaire to measure the number of adverse events participants had in early life, and a basic perceptual decision-making task in which we measured each participant’s decision threshold. If adverse events in early life have an effect on the basic process that underlies all decision-making, then we would expect to see a negative correlation between the number of adverse events in early life and the decision threshold adopted by participants in the perceptual decision-making task. However, as a multitude of other factors are likely to influence the decision, we would expect the correlation to be statistically reliable, though not necessarily strong (i.e., potentially a small-to-moderate correlation).

## Materials and Methods

### Participants and Procedure

Before data collection, we set an *a-priori* exclusion criterion for participants based on their accuracy, with the exclusions being automatically applied by data parsing scripts (i.e., we never assessed any of the excluded data). Participants who had less than 60% accuracy were considered to be close to chance performance, and therefore, unlikely to be performing the task correctly, and were excluded. Participants were undergraduate psychology students from the University of Newcastle participated in a large online survey for course credit. Upon arrival at the survey page, participants were provided with study information, and informed that the decision to progress through the study would be deemed as implied consent. Of the 242 participants that completed the study, only the results of the 181 participants, who completed both the life history survey and the perceptual decision-making task, are reported here^1^. After exclusions were applied (55 participants failed to make the 60% accuracy criterion), the data of 126 participants (101 female, mean age of 24.8) were remaining for analysis. Participants completed the experiment online at a time and location of their choosing, with survey questions presented through the Qualtrics survey platform and the perceptual decision-making task presented through purpose-built JavaScript code.

### Perceptual Decision-Making Task

Our perceptual decision-making task was the random dot kinematogram (Shadlen and Newsome, 1996; Evans and Brown, 2017), using the “white noise” algorithm (Pilly and Seitz, 2009). Participants were shown a cloud of 40 white dots (3 pixels each in diameter) on a black background and asked to decide whether the general movement of these dots was toward the top-left (“z” key) or top-right (“/” key) of the screen. The dots always remained within a circular area in the center of the screen, 150 pixels in diameter, and any dot that moved outside of this area was randomly re-placed within it. On each frame, 4 dots (i.e., 10%) were randomly selected to move √18 pixels in the correct direction (i.e., coherently), with all other dots being randomly re-placed within the area. Before each trial, a fixation cross was placed on the screen for a random exponentially distributed amount of time, with a mean of 700 ms, upper truncation of 4,800 ms, and an offset of 200 ms. After each trial, participants received feedback on whether their response was correct, and their response time and accuracy were recorded. Feedback was displayed for 500 ms, with an additional 1,000 ms timeout for errors, and a 1,000 ms inter-trial-interval. Participants completed 5 blocks of 40 trials each.

### Self-Report Distressing Life Events Scale

A list of potentially distressing life events was compiled containing items suggested by the Adverse Childhood Events study (Felitti et al., 1998; Centers for Disease Control and Prevention [CDC], 2018), health and wellbeing surveys conducted in the USA and Australia (Brim et al., 2000; AIHW, 2014; Australian Bureau of Statistics, 2014). Where appropriate, items were adapted to reflect immediacy of exposure—that is they were divided so that participants could note whether the event had happened to themselves, their family or household members, or witnessed outside the home. In some cases, this adaption created original items. The list was presented in as brief a form as possible so as to elicit information with a minimum of emotional engagement. Items were presented to pilot testers and modified according to feedback. In the final survey, participants were asked to indicate which, if any, of the following affected them during their lifetime. For those that applied, they were asked to indicate as best they could can remember, their age(s) at the time (e.g., 8, 12–14, 25–35). Final items include acute illness (yourself) (i.e., any health condition with an abrupt onset and duration, e.g., heart attack, pneumonia) and threatening/violent/abusive act(s) (against self) (see Supplementary Material for the full list, along with the source of the items).

### Subjective Units of Distress Scale

The Subjective Units of Distress Scale is a self-report measure of psychological distress. The scale is used (in both original and adapted form) in a range of settings including psychological interventions, (e.g., exposure therapy), (Wolpe, 1990; Kendall et al., 2015), hospital (e.g., monitoring patients, caregivers and families) (see for example Davis et al., 1994; Couper et al., 2013) and trauma research (see for example, Devilly and Spence, 1999; Douglas Bremner et al., 1999). Items range from No distress, totally relaxed (0) to Highest anxiety/distress you have ever felt (100). For the current study, participants were asked to indicate how they felt at the time each distressing event was experienced.

### Material Deprivation Scale

Financial stress before the age of 18 years was measured retrospectively using the 28-item Material Deprivation Scale (Knowles et al., unpublished). Participants rate their level of exposure on a five point likert scale from 1 (Never) to 5 (Always), where some items are reverse scored. The scale measures the subjective experience of material deprivation (e.g., missing out/disengagement, cash-flow problems) and hardship (Bray, 2001; Saunders et al., 2008), rather than household income and class status, typically included in measures of SES (Marks, 2007). This approach was selected as a means of distinguishing the experience of chronic and multiple stressors from more common and transient financial strains (Bray, 2001).

### Data Analysis

To estimate the level of early life adversity experienced by participants, we totalled the number of adverse childhood events that participants noted within the *self-report distressing life events scale*. Specifically, for each participant we created a *type of event* by *age* matrix for all potential events in the survey and the ages of 0–18, and made a binary classification for each cell of the matrix based on the answers to the scale (i.e., whether or not the participant experienced a specific adverse event at a specific age). We then took a sum of this matrix (*M* = 15, SD = 24), which provided our final estimate of the early life adversity. We also calculated a second early life adversity index, which we call Childhood Subjective Distress for clarity, where instead of having a binary classification we calculated distress (SUDS) per event per year for an overall measure of Childhood Subjective Distress (*M* = 52, SD = 88). Importantly, the pure number of distressing events may be misleading if some participants experienced either a few events that were each highly distressing, or a large number of events that were each not very distressing.

To estimate the decision urgency of each participant within the *perceptual decision-making task*, we used the Linear Ballistic Accumulator [LBA; (Brown and Heathcote, 2008)], a widely applied EAM. Importantly, the LBA contains an analytically solvable probability density function, allowing it to be efficiently fit to empirical data. The LBA contains five distinct parameters: the average (across trials) drift rate for each alternative (denoted *v*), the SD (across trials) in drift for each alternative (denoted *s*), the upper bound of a uniform distribution of starting evidence (used to represent random response biases across trials; denoted *A*), the decision threshold (denoted *b*), and the time dedicated to non-decision processes (denoted *t*_0_). As the exact amount of evidence required to trigger a decision technically varies between trials based on the random starting amount of evidence, we defined decision threshold of interest as the average amount of evidence required to trigger a decision across trials (Evans et al., 2017), which is simply *b*-(*A*/2). We estimated the parameters of the LBA for each participant using Bayesian parameter estimation, which provides a benefit over standard methods of estimation by capturing the uncertainty in the estimated parameter values. Our choice of non-hierarchical estimation over hierarchical estimation was due to hierarchical estimation creating parameter value “shrinkage” (i.e., all participants estimated values are drawn to one another), which although advantageous in many contexts, provides a bias toward detecting effects when placed in a “second-stage analysis,” such as a subsequent correlation (see Boehm et al., 2018 for a discussion). Proposals were generated via the differential evolution algorithm (Ter Braak, 2006; Turner et al., 2013), which we ran with 18 parallel chains for 4,000^2^ iterations each, with the first 2,000 iterations of each chain discarded as burn-in.

To assess the relationship between early life adversity and decision threshold, we used two different methods. The first method is one that is commonly applied when assessing the relationship between cognitive model parameters and other factors [performed via JASP; (JASP Team, 2018)], which involves taking a point-estimate measure of the cognitive parameter, and correlating it with the other method using standard correlation analyses. To obtain the point-estimate measure of decision-threshold, we took the median of the threshold posterior distribution for each participant, which is a robust measure of the central tendency. Although this first method is the one most commonly used to assess the relationship between cognitive parameters and other variables, the use of a point estimate from the posterior ignores the uncertainty in the estimated parameter value, meaning that the relationship between the variables is highly dependent on the specific point estimate taken for the cognitive parameter.

The second method involves estimating the decision threshold and the correlation between it and early life adversity simultaneously [i.e., a “joint-modeling” approach; (de Hollander et al., 2016)], which incorporates the uncertainty in parameter values into the estimated correlation, and the inferences made about whether a relationship between the factors exists. Importantly, the commonly applied method above has been shown to create a bias toward showing evidence for no effect (Boehm et al., 2018). In contrast, the “joint-modeling” approach has been advocated as the most complete and accurate method of assessing the relationship between cognitive model parameters and other factors (Matzke et al., 2017), but has rarely been applied due to the technical and computational burden associated with implementation. The exact prior distributions for the models can be seen in the Supplementary Material.

**FIGURE 1:**
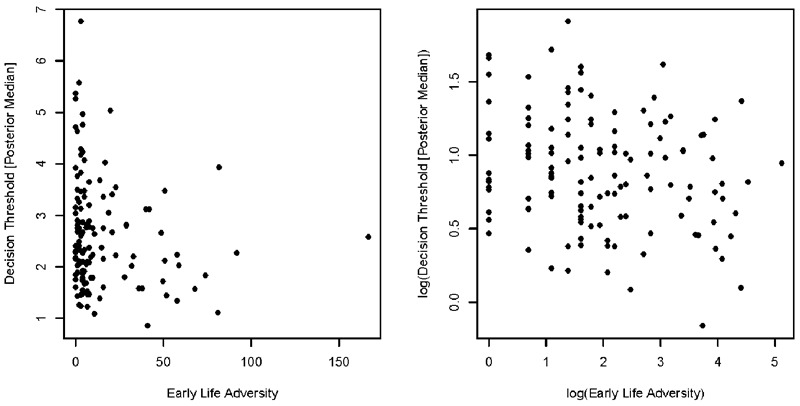
Scatterplot showing the relationship between our measure of early life adversity (*x*-axis) and posterior median estimate of decision threshold (*y*-axis), using the pre-transformed values (left) and log-transformed values (right). Although there appears to be some relationship between these two variables, the relationship appears to show some level of non-linearity, where participants with low levels of early life adversity often having extremely high thresholds, but this being less true of participants with greater levels of early life adversity. The natural logarithm transformation appears to remedy this issue, making the relationship fairly linear.

In all situations, we specified the alternative hypothesis as being a negative correlation—in accordance with our theoretical hypotheses—meaning that positive correlations reflect evidence in favor of the null model (or, in the case of frequentist statistics, a lack of evidence against the null; i.e., “one-tailed” analyses). As all variables showed a positive skew, and the relationship between the standard variable values did not appear to be linear (though still in monotonic pattern; Figure 1, left panel)—issues which could potentially invalidate parametric assumptions of our statistical analyses—we performed a natural logarithm (i.e., a base of Euler’s number) transformation to all variables^3^. We also performed parametric and non-parametric analyses on the standard variable values, and it should be noted that the estimated relationships were weaker in these cases, and that we did *not* decide upon the natural logarithm transformation *a-priori*.

To ensure that any relationship between early life adversity and decision threshold was not due to another potentially important factor in later-life decision-making, material deprivation, we also include partial correlation analyses with the variance attributed to material deprivation (*M* = 1.8, SD = 0.586) removed using the linear regression residuals method. We also develop a novel joint modeling analysis that estimates the partial correlation between early life adversity and decision threshold with the variance attributed to material deprivation removed.

Our study uses Bayes model comparison via Bayes factors to decide between the null (no relationship or positive correlation) and alternative (negative correlation) hypotheses. Importantly, Bayes factors compare the relative likelihood of the observed data given each model and can be directly interpreted as the amount of evidence for each hypothesis, avoiding many of the issues previously highlighted with standard frequentist analyses (i.e., null hypothesis significance testing). However, we also report the standard frequentist analyses for those who may be interested in the results of these significance tests.

## Results

**FIGURE 2:**
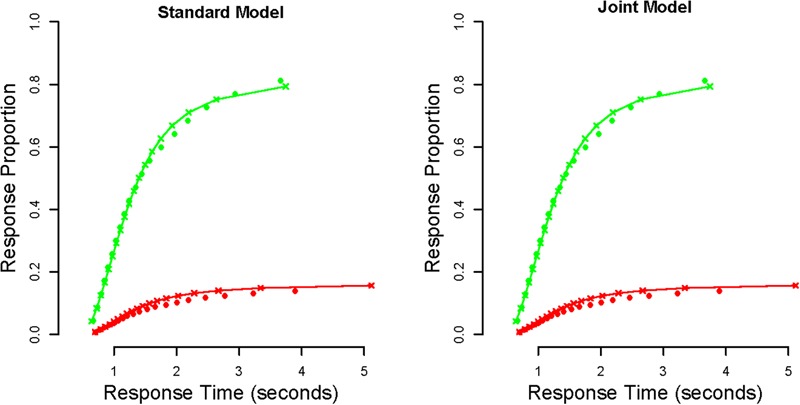
Defective cumulative density function plots that display the goodness-of-fit of the LBA to the perceptual decision-making data. The *x*-axis displays the response times and the *y*-axis displays the response proportions, with different points showing different response time quantiles. Red points display error responses, whereas green points display correct responses. Dots display the empirical data, and lines (with crosses) display the posterior model predictions. The left panel displays the group-averaged fits for the standard model (i.e., used to estimate the median threshold), and the right panel displays the group-averaged fits for the joint model. In both cases, the model appears to provide a generally good account of the data.

Our first assessment of whether early life adversity is related to decision urgency is simple decision-making involved correlating the natural logarithm of the median of the estimation posterior for decision threshold to the natural logarithm of our standard measure of early life adversity. In general, the LBA appeared to provide a good account of the data from the perceptual task (Figure 2), and an overall summary for each early life adversity index can be seen in Table 1 (Distressing Event by Age Matrix) and Table 2 (Childhood Subjective Distress). Pearson’s product-moment correlation coefficient showed a weak, negative correlation between the two variables (*r* = -0.21; Figure 1, right panel), which displays moderate evidence in favor of the alternative hypothesis (BF_10_ = 3.9) and a significant relationship between the variables (*p* = 0.008), suggesting that a relationship exists between early life adversity and decision threshold. Parametric and non-parametric (i.e., Kendall’s τ) correlations between the untransformed variables also showed a weak, negative correlation (*r* = -0.16; *r*_τ_ = -0.13), with the parametric correlations showing very weak evidence in favor of the alternative hypothesis (BF_10_ = 1.1) and a significant relationship (*p* = 0.036), and the non-parametric correlations showing weak evidence in favor of the alternative hypothesis (BF_10_ = 2.4) and a significant relationship (*p* = 0.017).

**Table 1:** Distressing event by age matrix.

	Age																	
Event Type	1	2	3	4	5	6	7	8	9	10	11	12	13	14	15	16	17	18
Acute Illness (Self)	1			1	1	2	1	2	2	3	1		1	4	5	4	3	4
Acute Illness (Household/Family Member)	2	2	2	3	4	1	2	2	3	5	4	5	3	3	5	10	5	12
Attempted Suicide ((Household/Family Member)	1			1		1				2	1	1	2	1	2	2	2	2
Attempted Suicide (Self)	1						1	1	1	1	1	2	1	6	6	5	4	2
Chronic Illness (Household/Family Member)	6	6	6	6	7	7	6	6	7	8	9	11	8	8	13	18	16	18
Chronic Illness (Self)								2	2	2	2	2	3	4	6	8	7	9
Criminal Act (by Self)	1													2	2	3	2	
Criminal Act (against Self)					1	1	1	1					1	1	1		2	1
Criminal Act by (Household/Family Member)						1	2	1	1	1	2	3	1	1	3	1		2
Criminal Act (against Household/Family Member)	1				3	2	2	2	3	3		1	2	2	2	1	2	
Criminal Act (outside home)	2	1	1	1	1	1	1	1	2	1	1	1	1	1	2	1	1	1
Death (Household/Family Member)				2	1	1	1	2	4	9	7	4	4	6	4	6	7	8
Death (Friend)	1												1	1	1	4	5	4
Disability (Household/Family Member)								1	1	1	1	1	1	1	1	1	1	1
Disability (Self)	2	2	2	2	3	2	3	4	3	3	3	3	3	3	3	4	5	4
Divorce/Separation (Parent/Guardian)	1	5	2	2	6	2		2	3	1		1	2	6	3	4	1	2
Employment Term	1											1	2		2	1	4	4
Gambling (Self)	1																	2
Gambling (Household/Family Member)				1	1	1	1	2	2	2	2	3	2	2	2	1	1	2
Imprison/Institut (Self)	1																	1
Imprison/Institut (Household/Family Member)			1	1			1		2		2	1	2	2	2	1	1	
Mental Illness (Self)					1	2	2	4	4	8	8	12	14	21	28	29	33	34
Mental Illness (Household/Family Member)	10	10	11	11	12	12	12	12	12	13	13	17	15	15	15	21	19	20
Serious Accident (Self)										1	1	1				1		
Serious Accident (Household/Family Member)				1			1	2		1	1	2	1	2	1	1		
Serious Accident (Outside Home)	1										1							1
Substance Abuse (Self)	1												2	2	3	5	6	5
Substance Abuse (Household/Family Member)	5	5	5	5	6	7	7	7	7	6	6	7	7	9	8	8	7	7
Violent/Threatening (against Self)			2	2	3	3	4	3	5	7	5	4	6	9	6	8	7	6
Violent/Threatening (against Household/Family Member)	2	1	1	3	2	4	6	5	5	6	5	5	4	4	3	4	5	4
Violent/Threatening (by Household/Family Member)	2	2	3	3	4	6	8	8	8	10	9	10	8	7	5	5	6	5
Violence (outside home)	1								1								1	
Verbal/Emotional (outside home)	3	3	3	5	7	9	10	10	13	12	14	17	19	23	20	22	19	16
Verbal/Emotional against (Self)	3	2	2	2	2	2	3	3	4	5	6	6	7	7	7	7	5	4
Verbal/Emotional against (Household/Family Member) (*N* = 126)									2	2	3	4	4	5	3	3	3	2

**Table 2:** Childhood subjective distress by age.

	Age		
Event Type	1	2	3	4	5	6	7	8	9	10	11	12	13	14	15	16	17	18
Acute Illness (Self)	4			2	4	6	5	9	7	14	3		5	41	42	46	41	47
Acute Illness (Household/Family Member)	50	50	50	52	61	50	54	60	63	86	111	116	86	86	103	144	117	121
Attempted Suicide((Household/Family Member)				1		4				40	35	35	35	35	40	41	9	7
Attempted Suicide (Self)							53	53	53	53	53	67	53	103	114	102	100	59
Chronic Illness (Household/Family Member)	353	353	353	353	357	356	353	353	356	364	412	417	358	351	426	473	318	322
Chronic Illness (Self)								50	50	50	50	50	56	78	111	127	89	94
Criminal Act (by Self)														11	22	31	27	
Criminal Act (against Self)					23	23	23	23						23	23		4	
Criminal Act by (Household/Family Member)						27	27	27	27	27	47	48	20	20	27	20		4
Criminal Act (against Household/Family Member)					41	40	40	40	32	31		3	7	21	21	2	7	
Criminal Act (outside home)	10	10	10	10	10	10	10	10	15	10	10	10	10	10	12	10	10	10
Death (Household/Family Member)				7	5	2	4	13	22	57	50	23	33	64	40	53	54	51
Death (Friend)													4	4	3	15	17	14
Disability (Household/Family Member)	130	130	130	130	133	130	177	180	177	177	177	177	177	177	177	187	191	187
Disability (Self)								43	43	43	43	43	43	43	43	43	43	43
Divorce/Separation (Parent/Guardian)		10	6	7	19	2		11	16	4		4	3	18	14	21	7	11
Employment Term												2	9		4	6	16	16
Gambling (Self)																		3
Gambling (Household/Family Member)				24	24	24	24	49	49	49	49	53	49	49	27	24	24	26
Imprison/Institut (Self)																		5
Imprison/Institut (Household/Family Member)			27	27			27		28		54	27	54	54	54	27	27	
Mental Illness (Self)					62	*66*	116	213	213	348	332	451	514	664	733	746	789	779
Mental Illness (Household/Family Member)	461	461	463	536	579	579	579	579	579	624	624	673	670	667	658	704	696	661
Serious Accident (Self)												3				3		
Serious Accident (Household/Family Member)				10			3	13		3	3	26	22	26	22	3		
Serious Accident (Outside Home)											4							1
Substance Abuse (Self)													30	30	46	62	63	62
Substance Abuse (Household/Family Member)	221	221	221	221	291	301	301	301	295	291	291	305	305	330	299	299	285	285
Violent/Threatening (against Self)				67	53	84	144	131	124	136	164	164	144	144	144	104	112	108
Violent/Threatening (against Household/Family Member)				67	53	84	144	131	124	136	164	164	144	144	144	104	112	108
Violent/Threatening (by Household/Family Member)	132	132	177	177	254	286	368	368	395	407	410	433	369	354	187	195	204	195
Violence (outside home)									5								6	
Verbal/Emotional (outside home)									5	6	9	31	31	53	47	47	46	42
Verbal/Emotional against (Self)	167	167	167	200	348	445	478	448	488	528	595	641	687	712	639	629	599	506
Verbal/Emotional against (Household/Family Member) (*N* = 126)	104	104	104	104	104	104	144	144	149	239	255	255	268	268	223	177	159	144

We also performed these assessments using the natural logarithm of our second index of early life adversity, Childhood Subjective Distress, which also showed a weak, negative correlation with the natural logarithm of median decision threshold (*r* = -0.22), with moderate evidence in favor of the alternative hypothesis (BF_10_ = 4.4) and a significant relationship (*p* = 0.007). This relationship held regardless of whether we only looked at distressing events that occurred in early childhood (i.e., ages 7 and under; *r* = -0.28, BF_10_ = 10.5, *p* = 0.003), or only distressing events that occurred in later childhood (i.e., ages 8–18; *r* = -0.2, BF_10_ = 2.8, *p* = 0.012), though the size of the effect and the evidence in favor of the alternative hypothesis varied. Lastly, all relationships held when applying partial correlations that removed the variance attributed to childhood material deprivation, with the standard early life adversity index (*r* = -0.23, BF_10_ = 5.8, *p* = 0.005), the Childhood Subjective Distress version with events from all ages (*r* = -0.23, BF_10_ = 5.4, *p* = 0.006), the Childhood Subjective Distress version with only events from 7 and under (*r* = -0.26, BF_10_ = 13.5, *p* = 0.002), and the Childhood Subjective Distress version with only events from 8 to 18 (*r* = -0.21, BF_10_ = 3.3, *p* = 0.01) all showing a weak, negative correlation that showed moderate-to-strong evidence for the alternative hypothesis and a significant relationship. Parametric and non-parametric correlations between the untransformed variables also showed weak, negative correlations for the Childhood Subjective Distress version with events from all ages (*r* = -0.17, BF_10_ = 1.3, *p* = 0.029; *r*_τ_ = -0.13, BF_10_ = 2.1, *p* = 0.018), the Childhood Subjective Distress version with only events from 7 and under (*r* = -0.13, BF_10_ = 0.6, *p* = 0.069; *r*_τ_ = -0.19, BF_10_ = 25.3, *p* = 0.003), and the Childhood Subjective Distress version with only events from 8 to 18 (*r* = -0.17, BF_10_ = 1.4, *p* = 0.027; *r*_τ_ = -0.12, BF_10_ = 1.5, *p* = 0.027), with the parametric correlations mostly showing very weak evidence for the alternative hypothesis and significant effects (and in the case of events from 7 and under, very weak evidence in favor of the null hypothesis and a non-significant effect), and the non-parametric correlations showing weak-to-strong evidence for the alternative hypothesis and significant effects. When partial correlations were used to remove the variance attributed to the untransformed material deprivation measure, a weak, negative correlation remained in all cases, though again with variable evidence across the standard early life adversity index (*r* = -0.16, BF_10_ = 1.1, *p* = 0.035; *r*_τ_ = -0.16, BF_10_ = 7.7, *p* = 0.004), the Childhood Subjective Distress version with events from all ages (*r* = -0.17, BF_10_ = 1.3, *p* = 0.029; *r*_τ_ = -0.15, BF_10_ = 4.1, *p* = 0.008), the Childhood Subjective Distress version with only events from 7 and under (*r* = -0.13, BF_10_ = 0.6, *p* = 0.077; *r*_τ_ = -0.19, BF_10_ = 38.6, *p* < 0.001), and the Childhood Subjective Distress version with only events from 8 to 18 (*r* = -0.17, BF_10_ = 1.4, *p* = 0.026; *r*_τ_ = -0.13, BF_10_ = 2.1, *p* = 0.018).

**FIGURE 3:**
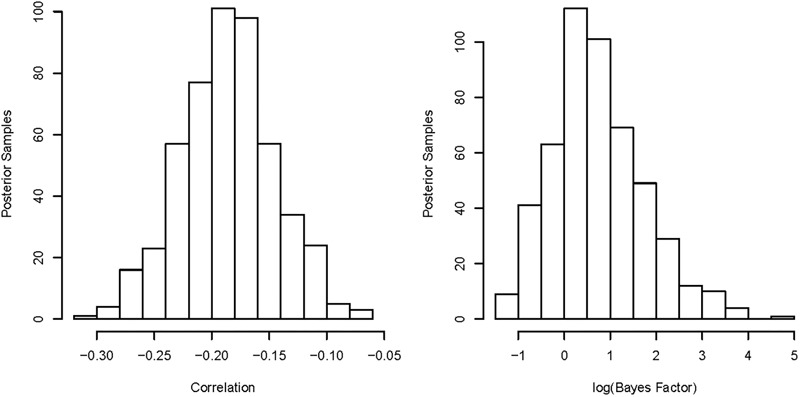
Left: histogram of the estimated correlations between the natural logarithms of our measure of early life adversity and decision threshold, with different correlations reflecting different samples of decision threshold from the posterior distribution. The chosen sample of decision threshold appears to have a large impact on the estimated correlation between the variables, ranging from –0.31 to –0.07, suggesting a great deal of uncertainty in the true correlation. Right: histogram of the estimated Bayes factors for evidence in favor of a correlation existing between our measure of early life adversity and decision threshold, with different Bays factors reflecting different samples of decision threshold from the posterior distribution. Note that we display the natural logarithm of the Bayes factor within this plot for ease of interpretation, where 0 indicates equal evidence for each hypothesis, 1 indicates that the alternative is *e* (i.e., Euler’s number) times more likely, and –1 indicates that the null is *e* times more likely. The chosen sample of decision threshold appears to have a large impact of the inference made about the correlation, ranging from moderate evidence in favor of the null, to strong evidence in favor of the alternative.

The analyses above seem to indicate a general uncertainty of whether or not an effect exists between early life adversity and decision threshold, and if an effect does exist, how strong the effect is. However, the analyses above all contain one key limitation; they ignore the uncertainty in the estimated decision threshold, which ignores additional information that can better inform the correlation analysis, and can even lead to a bias toward the null hypothesis (Boehm et al., 2018). A pictorial example of the impact of this uncertainty can be seen in Figure 3 (left) for the relationship between the log-transformed decision threshold and log-transformed standard index of early life adversity, which rather than taking the median of the threshold posterior, takes 500 random posterior samples and calculates the correlation and associated Bayes factor for each, which are then plotted as histograms [Figure 3 (right)]. As can be seen, when looking at the potential correlations between early life adversity and decision threshold across the range of posterior samples of decision threshold, the correlation ranges from -0.31 to -0.07. In addition, the Bayes factor ranges from moderate evidence in favor of the null to decisive evidence in favor of an effect. Importantly, this seems to indicate that analyses based on a point estimate of the posterior are incomplete, as the uncertainty in the threshold value can greatly change the estimated correlation, meaning that inferences can be highly dependent on which of many potentially sensible point estimates are chosen (e.g., median, mean, mode, etc.).

**FIGURE 4:**
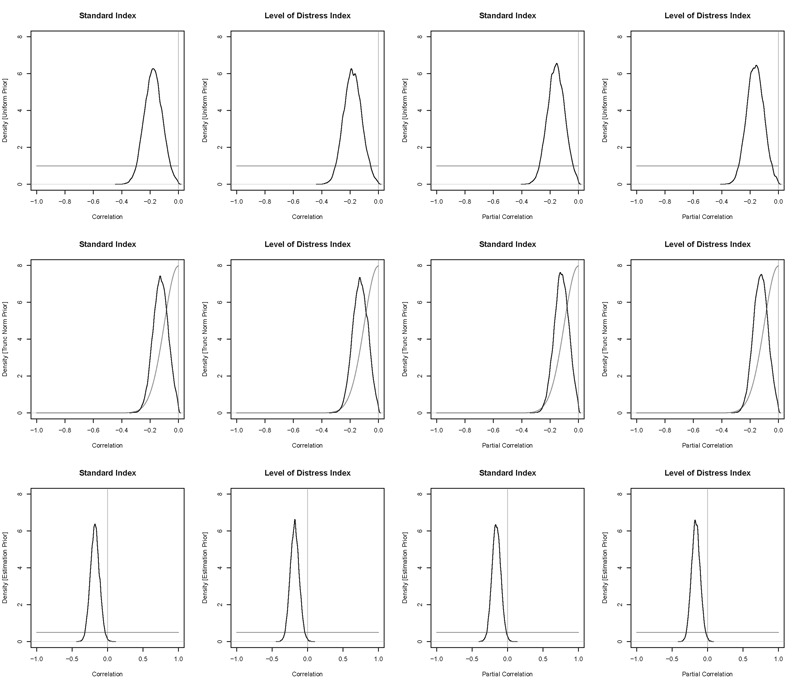
Density of the posterior correlation distribution (black) and the prior distribution (dark gray) from our joint modeling analysis. Panels in the first row display the analyses with the uniform prior truncated at 0 (which follows the “default” priors used for Bayesian correlation analysis), panels in the second row display the analyses with the truncated normal prior, and panels in the third row display the analyses with the “estimation prior” (i.e., uniform with no truncated at 0). The first column displays the standard ELA index, and the second column displays the ELA index that accounts for the level of distress caused by the events. The third and fourth rows display the same respective indexes, though with the partial correlations that remove the variance attributed to SES. The light gray vertical line reflects the fixed point of 0, where the density of the posterior and the prior are compared to computed the Savage-Dickey ratio estimate of the Bayes.

To take into account this uncertainty, and hopefully provide a more accurate and conclusive answer, we took a “joint-modeling” approach (de Hollander et al., 2016) to measuring the correlation between these factors, which has been advocated by Boehm et al. (2018) and Matzke et al. (2017), and has been applied in a similar manner in Evans et al. (2017) and Evans et al. (2018b). This involved simultaneously estimating the decision threshold and the correlation between it and early life adversity, using a Bayesian hierarchical model and a bivariate normal distribution [in the language R, (R Core Team, 2013)] at the hierarchical level for these two factors. Note that we only performed these analyses for the log-transformed data, as performing these joint modeling analyses are computationally taxing, especially given that we perform 12 different variants of the analysis for robustness. In order to be consistent with the previous correlations, we assumed a uniform prior on the correlation parameter, which when compared to the estimated correlation posterior (Figure 4; first row) using the Savage-Dickey ratio (Wagenmakers et al., 2010) found strong evidence in favor of a negative relationship between early life adversity and decision threshold (BF_10_ = 12.1)^4^. However, it should also be noted that the uniform prior is known to be a “conservative” prior in the calculation of Bayes factors (Lindley, 1957; Rouder et al., 2012), meaning that it is harder to find evidence for the alternative hypothesis (and easier to find evidence for the null^5^). The intuitive reason for the uniform prior being conservative is the equal density that it assigns to all possible values across the entire parameter space makes the alternative hypothesis more general, and therefore, more flexible, making it less likely to be preferred unless there is strong evidence in favor of it within the data. Therefore, the evidence in favor of an effect with a uniform prior provides some “lower bound” on the potential evidence in favor of an effect in our joint modeling assessment. In order to gain an “upper bound” on the potential evidence in favor of an effect in our joint modeling assessment, we require a less conservative prior that contains more peaked density over specific values, while remaining reasonable given that we have little prior knowledge of the expected relationship between these two variables (i.e., a prior that is not overly narrow). Our choice of prior for this upper bound was a truncated normal distribution with a mean of 0 and a SD of 0.1 (Figure 4, second row), which is a simple distribution that is much more peaked than the uniform distribution, while still assigning reasonable probability to larger correlation values. While using the truncated normal prior, we found strong evidence in favor of a negative relationship (BF_10_ = 24.52)^6^, meaning that our joint modeling analysis seems to suggest some evidence in favor of a relationship between early life adversity and decision threshold, albeit a somewhat small effect.

For robustness, we also performed several additional joint modeling analyses. Firstly, as our previous analyses restricted the prior to only contain negative correlation values, in line with our prediction of a negative relationship between the variables, we also estimated the joint model with a completely uninformed prior (i.e., a uniform distribution from -1 to 1 on the correlation; we label this the “estimation prior” below) to obtain a less restricted estimate of the posterior distribution. Importantly, estimation has recently been advocated over hypothesis testing (Kruschke, 2011; Kruschke and Liddell, 2018), and these posterior distributions can then be used as prior distributions for future work (Lindley, 1972; Wagenmakers et al., 2010). The estimated correlation posterior can be seen in Figure 4 (third row), with the vast majority of the density being over negative values (credible intervals = [-0.3, -0.05]), and the distribution being centered on a correlation of -0.18. As would be expected from this more general prior, the Savage-Dickey ratio provided weaker (moderate) evidence than before in favor of an effect (BF_10_ = 3.57), though our aim of this joint model analysis was to provide an estimate of the posterior distribution, rather than a hypothesis test. Secondly, we also performed the joint model analysis on our Childhood Subjective Distress version of early life adversity, finding strong evidence for a negative relationship with the uniform prior (BF_10_ = 11.52) and the truncated normal prior (BF_10_ = 25.6). The estimation prior suggested that the correlation was almost certainly negative (credible intervals = [-0.3, -0.05]), centered on -0.18, and provided moderate evidence in favor of an effect (BF_10_ = 3.31). Lastly, we performed all of these analyses with a partial correlation joint model, which removed the variance attributed to childhood material deprivation. Both indexes of early life adversity came to the same general conclusions as before, with the standard index finding moderate evidence for a negative relationship with the uniform prior (BF_10_ = 6.09) and strong evidence with the truncated normal prior (BF_10_ = 18.91), and the estimation prior showing credible values of [-0.28, -0.03] and weak evidence in favor of an effect (BF_10_ = 1.65). The Childhood Subjective Distress version found moderate evidence for a negative relationship with the uniform prior (BF_10_ = 8.48) and strong evidence with the truncated normal prior (BF_10_ = 28.11), and the estimation prior showed credible values of [-0.28, -0.04] and weak evidence in favor of an effect (BF_10_ = 2.33). Overall, our joint modeling analyses appear to show weak-strong evidence in favor of a weak relationship between early life adversity and decision threshold.

## Discussion

Informed by both cognitive and evolutionary theory, we predicted that early life adversity would have an impact upon the decision process used in later life decision-making. More specifically, we expected that the number of distressing events experienced in childhood would be related to the level of urgency displayed in making simple decisions (a random dot kinematogram task), measured through the decision threshold in an EAM, the Linear Ballistic Accumulator, where those who experienced more distressing life events would be more urgent in their decision strategy. In general, our findings were mixed when using simple correlation analyses, where most analyses showed weak evidence in favor of a negative relationship between these factors, though with a great deal of inconsistency between a range of different potential assumptions. However, a key limitation of these simple correlation analyses is that they fail to account for the uncertainty in the estimated decision threshold value, using the median of an entire posterior of potential values. When performing a joint modeling analysis—advocated by Boehm et al. (2018) and Matzke et al. (2017) and similar to Evans et al. (2017) and Evans et al. (2018b), but still relatively novel due to the difficulty in implementation—we found weak-strong evidence in favor of the negative relationship between these two variables, though the overall strength of the relationship was fairly weak. Therefore, based on the final joint modeling analysis being the most complete, we believe that our study shows evidence, albeit limited, for a weak relationship between early life adversity and the level of urgency in the basic decision process.

We believe that our work provides an extremely important first step in answering a novel theoretical question that has a substantial degree of applied relevance. Although we discuss a large body of research in the introduction that convergences on the theoretical similarity of the concept of “urgency” in a life-history context and a decision-making context, no previous studies (to the best of our knowledge) have previously assessed whether a relationship between early life adversity and decision urgency exists. Importantly, if a relationship between these factors exists, then this would suggest that experiencing early life adversity impacts upon the basic algorithmic process that underlies decision-making, resulting in those who experience adversity becoming universally more urgent in their decision strategies, regardless of the consequences, and regardless of the context. However, the evidence within our study for a relationship between early life adversity and decision urgency is limited, and therefore, strong inferences about whether or not an effect exists should not be drawn from our work. Instead, we believe a great deal of future research is needed to attempt to establish whether this effect and/or other potentially related effects exist, and whether the impact upon decision strategy is “universal” across different types of decisions, which could be aided by the use of a battery of perceptual tasks with a latent variable approach to assess the generalizability of the effect across tasks [e.g., (Schmiedek et al., 2007)]. In addition, we believe that there are several potentially interesting extensions from this study that could further test the existence of the relationship between early life adversity and decision urgency. For example, a potential extension would be to incentivise urgent and/or cautious responding with different types of rewards, which may create a situation where participants have more motivation to decide between urgent and cautious strategies. It should also be noted that our *a-priori* exclusion criteria, while aimed at excluding participants who were not performing the task properly (i.e., data contaminants), may have actually removed participants who were just extremely urgent, and that future research of this topic should aim to better distinguish between these two types of participants to ensure that data sets are not incorrectly censored.

As mentioned at the end of the introduction, we did not expect the relationship between early life adversity and decision urgency—if it existed—to be large. Importantly, we believed that early life adversity would only be one small part of what determines how urgent people are in their decision strategies, limiting the size in any relationship observed between these variables. Previous research has indicated a plethora of factors that influence the decision urgency adopted, such as task instructions (Rae et al., 2014), impending deadlines (Evans et al., 2018a), practice (Evans and Hawkins, 2019), white matter integrity (Forstmann et al., 2010), personality (Evans et al., 2017), and genetics (Evans et al., 2018a), just to name a few, with each explaining small to moderate portions of variability in decision urgency. Therefore, any relationship between two factors as distant from one another as early life adversity and the decision urgency would likely prove to be relatively small, as we appear to have observed within our study. However, we believe that the small size of the effect does not take away from its applied importance in understanding how early life adversity effects later life decision-making.

Lastly, when looking at the relationship between early life adversity and decision threshold, there appeared to be a strong non-linear, though monotonic, relationship between the variables. Specifically, those who experienced a small amount of early-life adversity appeared to have a higher probability of having less decision urgency, and those who experienced a larger amount of early-life adversity appeared to have a lower probability of having less decision urgency. Therefore, although we found a linear correlation between these two factors, future research should potentially explore whether the nature of the relationship between these two factors is more discrete. For example, future studies could potentially compare groups with known high levels of early life adversity to those with low levels of early life adversity (or random members of the population), and assess whether the decision urgency displayed in those with high levels of early life adversity appears to be systematically higher than those without. These more discrete distinctions may prove easier to explore, while still retaining the important applied implications discussed earlier.

## Ethics Statement

Approved by the University of Newcastle Human Research Ethics Committee.

## Author Contributions

All authors conceptualized the study, developed the methodology, and were involved in the reviewing and editing of the manuscript. JK and NE performed the data curation and wrote the original draft of the manuscript. JK performed the investigation, project administration, and obtained the resources for conducting the survey. NE wrote the software, performed the formal analysis, and visualized the data. DB provided supervision for the project.

## Conflict of Interest Statement

The authors declare that the research was conducted in the absence of any commercial or financial relationships that could be construed as a potential conflict of interest.

## Footnotes

1 The measures reported used here is a subset of data collected for a larger project, but only the data relevant to our hypothesis (i.e., the Distressing Life Events Scale) are included here. Another 15 participants completed the RDK, but their ID codes did not correspond with the ID codes of those who had completed the Distressing Life Events scale.

2 As the joint modeling assessment relied on the full posterior distribution, and not just a point estimate, we used 5,000 iterations each for the joint modeling assessment.

3 For the measurements of early life adversity and socio-economic status, we first added 1 to the measurements before taking the natural logarithm, in order to avoid infinite value problems caused by taking the logarithm of 0.

4 However, note that when the analysis was performed with the untransformed variable values (using a truncated normal distribution), there was only moderate evidence in favor of an effect, BF_10_ = 5.

5 It should also be noted that this is equivalent to the default prior used in JASP, meaning that our previous analyses were similarly conservative.

6 However, note that when the analysis was performed with the untransformed variable values (using a truncated normal distribution), there was only moderate evidence in favor of an effect, BF_10_ = 5.

## References

[B1] AIHW (2014). *Australia’s Health 2014.* Canberra: AIHW.

[B2] Anand P. Lea S. (2011). The psychology and behavioural economics of poverty. *J. Econ. Psychol.* 32 284–293. 10.1016/j.joep.2010.11.004

[B3] Australian Bureau of Statistics (2014). *General Social Survey (GSS) Household Survey Questionnaire.* Available at: http://www.ausstats.abs.gov.au/ausstats/subscriber.nsf/0/DE47CCADC083E9BBCA257E7000154993/\protect\T1\textdollarFile/41590do001_household_questionnaire.pdf

[B4] Bateson P. Gluckman P. Hanson M. (2014). The biology of developmental plasticity and the predictive adaptive response hypothesis. *J. Physiol.* 592 2357–2368. 10.1113/jphysiol.2014.271460 24882817 PMC4048093

[B5] Belsky J. Steinberg L. Draper P. (1991). Childhood experience, interpersonal development, and reproductive strategy: and evolutionary theory of socialization. *Child Dev.* 62 647–670. 10.1111/1467-8624.ep9109162242 1935336

[B6] Bertrand M. Mullainathan S. Shafir E. (2016). A behavioral-economics view of poverty. *Am. Econ. Rev.* 94 419–423. 10.1257/0002828041302019

[B7] Boehm U. Marsman M. Matzke D. Wagenmakers E.-J. (2018). On the importance of avoiding shortcuts in applying cognitive models to hierarchical data. *Behav. Res. Methods* 50 1614–1631. 10.3758/s13428-018-1054-3 29949071 PMC6096647

[B8] Bray J. R. (2001). *Hardship in Australia: An Analysis of Financial Stress Indicators in the 1998-1999 Australian Bureau of Statistics Household Expenditure Survey.* Available at: http://ssrn.com/abstract=1729046

[B9] Brim O. G. Bumpass L. L. Cleary P. D. Featherman D. L. Hazzard W. R. Kessler R. C. (2000). *National Survey of Midlife Development in the United States (MIDUS), 1995–1996.* Ann Arbor, MI: Inter-university Consortium for Political and Social Research.

[B10] Brown S. D. Heathcote A. (2008). The simplest complete model of choice response time: linear ballistic accumulation. *Cognit. Psychol.* 57 153–178. 10.1016/j.cogpsych.2007.12.002 18243170

[B11] Brumbach B. H. Figueredo A. J. Ellis B. J. (2009). Effects of harsh and unpredictable environments in adolescence on development of life history strategies. *Hum. Nat.* 20 25–51. 10.1007/s12110-009-9059-3 20634914 PMC2903759

[B12] Centers for Disease Control and Prevention [CDC] (2018). *Adverse Childhood Experiences Study.* Available at https://www.cdc.gov/violenceprevention/acestudy/

[B13] Chisholm J. S. (1993). Death, hope, and sex: life-history theory and the development of reproductive strategies. *Curr. Anthropol.* 34 1–24. 10.1086/204131

[B14] Chisholm J. S. (1999). *Death, Hope, and Sex: Steps to an Evolutionary Ecology of Mind and Morality.* Cambridge, MA: Cambridge University Press 10.1017/CBO9780511605932

[B15] Cole L. C. (1954). The population consequences of life history phenomena. *Q. Rev. Biol.* 29 103–137. 10.1086/400074 13177850

[B16] Couper J. W. Pollard A. C. Clifton D. A. (2013). Depression and cancer. *Med. J. Aust.* 199 13–16. 10.5694/MJA12.10522 25370277

[B17] Danese A. McEwen B. S. (2012). Adverse childhood experiences, allostasis, allostatic load, and age-related disease. *Physiol. Behav.* 106 29–39. 10.1016/j.physbeh.2011.08.019 21888923

[B18] Dańko M. J. Burger O. Argasiński K. Kozłowski J. (2018). Extrinsic mortality can shape life-history traits, including senescence. *Evol. Biol.* 45 395–404. 10.1007/s11692-018-9458-7 30459480 PMC6223763

[B19] Davis T. M. Maguire T. O. Haraphongse M. Schaumberger M. R. (1994). Undergoing cardiac catheterization: the effects of informational preparation and coping style on patient anxiety during the procedure. *Heart Lung* 23 140–150. 8206772

[B20] de Hollander G. Forstmann B. U. Brown S. D. (2016). Different ways of linking behavioral and neural data via computational cognitive models. *Biol. Psychiatry Cogn. Neurosci. Neuroimaging* 1 101–109. 10.1016/j.bpsc.2015.11.004 29560872

[B21] Del Giudice M. (2014). Early stress and human behavioral development: emerging evolutionary perspectives. *J. Dev. Orig. Health Dis.* 5 270–280. 10.3390/s151229838 24965133

[B22] Del Giudice M. Ellis B. J. Shirtcliff E. A. (2011). The adaptive calibration model of stress responsivity. *Neurosci. Biobehav. Rev.* 35 1562–1592. 10.1016/j.neubiorev.2010.11.007 21145350 PMC3068241

[B23] Denver R. J. Middlemis-Maher J. (2010). Lessons from evolution: developmental plasticity in vertebrates with complex life cycles. *J. Dev. Orig. Health Dis.* 1 282–291. 10.1017/S2040174410000279 25141931

[B24] Devilly G. J. Spence S. H. (1999). The relative efficacy and treatment distress of EMDR and a cognitive-behavior trauma treatment protocol in the amelioration of posttraumatic stress disorder. *J. Anxiety Disord.* 13 131–157. 10.1016/S0887-6185(98)00044-9 10225505

[B25] Douglas Bremner J. Narayan M. Staib L. H. Southwick S. M. McGlashan T. Charney D. S. (1999). Neural correlates of memories of childhood sexual abuse in women with and without posttraumatic stress disorder. *Am. J. Psychiatry* 156 1787–1795. 10.1176/ajp.156.11.1787 10553744 PMC3233772

[B26] Edwards V. J. Holden G. W. Felitti V. J. Anda R. F. (2003). Relationship between multiple forms of childhood maltreatment and adult mental health in community respondents: results from the adverse childhood experiences study. *Am. J. Psychiatry* 160 1453–1460. 10.1176/appi.ajp.160.8.1453 12900308

[B27] Evans N. J. Brown S. D. (2017). People adopt optimal policies in simple decision-making, after practice and guidance. *Psychon. Bull. Rev.* 24 597–606. 10.3758/s13423-016-1135-1 27562760

[B28] Evans N. J. Hawkins G. E. (2019). When humans behave like monkeys: feedback delays and extensive practice increase the efficiency of speeded decisions. *Cognition* 184 11–18. 10.1016/j.cognition.2018.11.014 30553935

[B29] Evans N. J. Hawkins G. E. Brown S. D. (2018a). The role of passing time in decision-making. *PsyArXi* [Preprint] 10.31234/osf.io/3wq6g 31180704

[B30] Evans N. J. Steyvers M. Brown S. D. (2018b). Modeling the covariance structure of complex datasets using cognitive models: an application to individual differences and the heritability of cognitive ability. *Cogn. Sci.* 42 1925–1944. 10.1111/cogs.12627 29873105

[B31] Evans N. J. Rae B. Bushmakin M. Rubin M. Brown S. D. (2017). Need for closure is associated with urgency in perceptual decision-making. *Mem. Cognit.* 45 1193–1205. 10.3758/s13421-017-0718-z 28585159

[B32] Fawcett T. W. McNamara J. M. Houston A. I. (2012). When is it adaptive to be patient? A general framework for evaluating delayed rewards. *Behav. Process.* 89 128–136. 10.1016/j.beproc.2011.08.015 21920413

[B33] Felitti V. J. Anda R. F. Nordenberg D. Williamson D. F. Spitz A. M. Edwards V. (1998). Relationship of childhood abuse and household dysfunction to many of the leading causes of death in adults. *Am. J. Prev. Med.* 14 245–258. 10.1016/S0749-3797(98)00017-8 9635069

[B34] Forstmann B. U. Anwander A. Schäfer A. Neumann J. Brown S. Wagenmakers E.-J. (2010). Cortico-striatal connections predict control over speed and accuracy in perceptual decision making. *Proc. Natl. Acad. Sci. U.S.A.* 107 15916–15920. 10.1073/pnas.1004932107 20733082 PMC2936628

[B35] Griskevicius V. Ackerman J. M. Cantú S. M. Delton A. W. Robertson T. E. Simpson J. A. (2013). When the economy falters, do people spend or save? responses to resource scarcity depend on childhood environments. *Psychol. Sci.* 24 197–205. 10.1177/0956797612451471 23302295

[B36] Griskevicius V. Tybur J. M. Delton A. W. Robertson T. E. (2011). The influence of morality and socioeconomic status on risk and delayed rewards: a life history theory approach. *J. Pers. Soc. Psychol.* 100 1015–1026. 10.1037/a00224030. 21299312 PMC3298774

[B37] Hemmingsson E. Johansson K. Reynisdottir S. (2014). Effects of childhood abuse on adult obesity: a systematic review and meta-analysis. *Obes. Rev.* 15 882–893. 10.1111/obr.12216 25123205

[B38] Hill E. M. Blow F. C. Young J. P. Singer K. M. (1994). Family history of alcoholism and childhood adversity: joint effects on alcohol consumption and dependence. *Alcohol. Clin. Exp. Res.* 18 1083–1090. 10.1111/j.1530-0277.1994.tb00085.x 7847588

[B39] Hill E. M. Jenkins J. Farmer L. (2008). Family unpredictability, future discounting, and risk taking. *J. Soc. Econ.* 37 1381–1396. 10.1016/j.socec.2006.12.081

[B40] Hill E. M. Newlin D. B. (2002). Evolutionary approaches to addiction. *Addiction* 97 375–379. 10.1046/j.1360-0443.2002.t01-1-00057.x 11964054

[B41] JASP Team (2018). *JASP (Version 0.8.6).*

[B42] Kendall P. C. Robin J. A. Hedike K. A. Sueveg C. Flannery-Schroeder E. Gosch E. (2015). Considering CBT with anxious youth? Think exposures. *Cogn. Behav. Pract.* 12 136–148. 10.1016/S1077-7229(05)80048-3

[B43] Kidd C. Palmeri H. Aslin R. N. (2013). Rational snacking: young children’s decision-making on the marshmallow task is moderated by beliefs about environmental reliability. *Cognition* 126 109–114. 10.1016/j.cognition.2012.08.004 23063236 PMC3730121

[B44] Koenen K. C. Roberts A. L. Stone D. M. Dunn E. C. (2010). “The epidemiology of early childhood trauma,” in *The Impact of Early Life Trauma on Health and Disease: The Hidden Epidemic* ed. Lanius R. A. (Cambridge: Cambridge University Press) 13–24. 10.1017/CBO9780511777042.003

[B45] Kruger D. J. Reischl T. Zimmerman M. A. (2008). Time perspective as a mechanism for functional developmental adaptation. *J. Soc. Evol. Cult. Psychol.* 2 1–22. 10.1037/h0099336 15845347

[B46] Kruschke J. K. (2011). Bayesian assessment of null values via parameter estimation and model comparison. *Perspect. Psychol. Sci.* 6 299–312. 10.1177/1745691611406925 26168520

[B47] Kruschke J. K. Liddell T. M. (2018). The Bayesian new statistics: hypothesis testing, estimation, meta-analysis, and power analysis from a Bayesian perspective. *Psychon. Bull. Rev.* 25 178–206. 10.3758/s13423-016-1221-4 28176294

[B48] Lindley D. V. (1957). A statistical paradox. *Biometrika* 44 187–192. 10.1093/biomet/44.1-2.187

[B49] Lindley D. V. (1972). *Bayesian Statistics, a Review* 6th Edn Vol. 2 Montpelier: Society for Industrial and Applied Mathematics 10.1137/1.9781611970654

[B50] Lovallo W. R. Farag N. H. Sorocco K. H. Cohoon A. J. Vincent A. S. (2012). Lifetime adversity leads to blunted stress axis reactivity: studies from the Oklahoma family health patterns project. *BPS* 71 344–349. 10.1016/j.biopsych.2011.10.018 22112928 PMC3264696

[B51] Marks G. N. (2007). Income poverty, subjective poverty and financial stress. *Paper Presented at the Australian Government Social Policy Research* Paper No. 29 Canberra 10.2139/ssrn.1728587

[B52] Mathot K. J. Frankenhuis W. E. (2018). Models of pace-of-life syndromes (POLS): a systematic review. *Behav. Ecol. Sociobiol.* 72:41 10.1007/s00265-018-2459-9

[B53] Matzke D. Ly A. Selker R. Weeda W. D. Scheibehenne B. Lee M. D. (2017). Bayesian inference for correlations in the presence of measurement error and estimation uncertainty. *Collabra Psychol.* 3:25 10.1525/collabra.78

[B54] Mersky J. P. Topitzes J. Reynolds A. J. (2013). Impacts of adverse childhood experiences on health, mental health, and substance use in early adulthood: a cohort study of an urban, minority sample in the U.S. *Child Abuse Neglect* 37 917–925. 10.1016/J.CHIABU.2013.07.011 23978575 PMC4090696

[B55] Mischel W. Ayduk O. Berman M. G. Casey B. J. Gotlib I. H. Jonides J. (2011). “Willpower” over the life span: decomposing self-regulation. *Soc. Cogn. Affect. Neurosci.* 6 252–256. 10.1093/scan/nsq081 20855294 PMC3073393

[B56] Mittal C. Griskevicius V. Simpson J. A. Sung S. Young E. S. (2015). Cognitive adaptations to stressful environments: when childhood adversity enhances adult executive function. *J. Pers. Soc. Psychol.* 109 604–621. 10.1037/pspi0000028 26414842

[B57] Nettle D. Frankenhuis W. E. (2019). The evolution of life history theory: bibliometric analysis of an interdisciplinary research area. *bioRxiv* [Preprint] 10.1101/510826 PMC6452081 30914012

[B58] Nettle D. Frankenhuis W. E. Rickard I. J. (2013). The evolution of predictive adaptive responses in human life history. *Proc. R. Soc. B Biol. Sci.* 280:20131343. 10.1098/rspb.2013.1343 23843395 PMC3730599

[B59] Paál T. Carpenter T. Nettle D. (2015). Childhood socioeconomic deprivation, but not current mood, is associated with behavioural disinhibition in adults. *PeerJ* 3:e964. 10.7717/peerj.964 26020014 PMC4435446

[B60] Pilly P. K. Seitz A. R. (2009). What a difference a parameter makes: a psychophysical comparison of random dot motion algorithms. *Vis. Res.* 49 1599–1612. 10.1016/J.VISRES.2009.03.019 19336240 PMC2789308

[B61] R Core Team (2013). *R: A Language and Environment for Statistical Computing.* Vienna: R Foundation for Statistical Computing.

[B62] Rae B. Heathcote A. Donkin C. Averell L. Brown S. (2014). The hare and the tortoise: emphasizing speed can change the evidence used to make decisions. *J. Exp. Psychol. Learn. Mem. Cogn.* 40 1226–1243. 10.1037/a0036801 24797438

[B63] Ratcliff R. (1978). *A Theory of Memory Retrieval. Psychological Review* Vol. 8 Available at: http://citeseerx.ist.psu.edu/viewdoc/download? 10.1037/0033-295X.85.2.59 3406246

[B64] Roff D. A. (1992). *The Evolution of Life Histories: Theory and Analysis.* Available at: https://www.springer.com/gp/book/9780412023910

[B65] Rouder J. N. Morey R. D. Speckman P. L. Province J. M. (2012). Default Bayes factors for ANOVA designs. *J. Math. Psychol.* 56 356–374. 10.1016/J.JMP.2012.08.001

[B66] Saunders P. Naidoo Y. Griffiths M. (2008). Towards new indicators of disadvantage: deprivation and social exclusion in Australia. *Aust. J. Soc. Issues* 43 175–194. 10.1002/j.1839-4655.2008.tb00097.x

[B67] Schmiedek F. Oberauer K. Wilhelm O. Süß H.-M. Wittmann W. W. (2007). Individual differences in components of reaction time distributions and their relations to working memory and intelligence. *J. Exp. Psychol. Gen.* 136 414–429. 10.1037/0096-3445.136.3.414 17696691

[B68] Shadlen M. N. Newsome W. T. (1996). Motion perception: seeing and deciding. *Proc. Natl. Acad. Sci. U.S.A.* 93 628–633. 10.1073/pnas.93.2.628 8570606 PMC40102

[B69] Stearns S. C. (1976). Life-history tactics: a review of the ideas. *Q. Rev. Biol.* 51 3–47. 10.1086/409052 778893

[B70] Ter Braak C. J. (2006). A markov chain monte carlo version of the genetic algorithm differential evolution: easy bayesian computing for real parameter spaces. *Stat. Comput.* 16 239–249. 10.1007/s11222-006-8769-1

[B71] Turner B. M. Sederberg P. B. Brown S. D. Steyvers M. (2013). A method for efficiently sampling from distributions with correlated dimensions. *Psychol. Methods* 18 368–384. 10.1037/a0032222 23646991 PMC4140408

[B72] van Ravenzwaaij D. Brown S. Wagenmakers E.-J. (2011). An integrated perspective on the relation between response speed and intelligence. *Cognition* 119 381–393. 10.1016/J.COGNITION.2011.02.002 21420077

[B73] Wagenmakers E.-J. Lodewyckx T. Kuriyal H. Grasman R. (2010). Bayesian hypothesis testing for psychologists: a tutorial on the Savage–Dickey method. *Cognit. Psychol.* 60 158–189. 10.1016/J.COGPSYCH.2009.12.001 20064637

[B74] Wolpe J. (1990). *Practice of Behavior Therapy* Vol. 4a. Available at: https://books.google.com.au/books/about/The_Practice_of_Behavior_Therapy.html?id=FTEpAAAAYAAJ

