# Supplementary material for: Some Evidence for an Association Between Early Life Adversity and Decision Urgency
Source: Front Psychol. 2019 Feb 11;10:243. doi: 10.3389/fpsyg.2019.00243 (PMC6377396; doi:10.3389/fpsyg.2019.00243)
Supplement: Supplementary file 1 [file Data_Sheet_2.PDF]

Individual Subjects Model

$$
\begin{aligned}
&Prior\ distributions: \\
&\qquad v_c \sim TN(3, 3, 0, Inf) \\
&\qquad v_e \sim TN(2, 3, 0, Inf) \\
&\qquad sv_e \sim TN(1, 1, 0, Inf) \\
&\qquad A \sim TN(2, 2, 0, Inf) \\
&\qquad b - A \sim TN(2, 2, 0, Inf) \\
&\qquad t_0 \sim TN(0.5, 0.5, 0, Inf)
\end{aligned}
$$

where TN is the truncated normal distribution with parameters $TN($*mean*, *standard deviation*, *lower truncation*, *upper truncation*$)$, the subscripts c and e represent the accumulators for correct and error responses respectively, and sv_c is fixed to 1 to satisfy a scaling property within the model.

Joint Model

$$
\begin{aligned}
Group\ level: & & \\
v_c &\sim& TN(\mu_{v_c}, \sigma_{v_c}, 0, Inf) \\
v_e &\sim& TN(\mu_{v_e}, \sigma_{v_e}, 0, Inf) \\
sv_e &\sim& TN(\mu_{sv_e}, \sigma_{sv_e}, 0, Inf) \\
A &\sim& TN(\mu_A, \sigma_A, 0, Inf) \\
t_0 &\sim& TN(\mu_{t_0}, \sigma_{t_0}, 0, Inf) \\
\begin{bmatrix} log(b - \frac{A}{2}) \\ log(ELA) \end{bmatrix} &\sim& BN\left(\begin{bmatrix} \mu_b \\ \mu_{ELA} \end{bmatrix}, \begin{bmatrix} \sigma_b^2 & \rho\sigma_b\sigma_{ELA} \\ \rho\sigma_b\sigma_{ELA} & \sigma_{ELA}^2 \end{bmatrix}\right]) \\
Prior\ distributions: & & \\
\mu_{v_c} &\sim& TN(3, 3, 0, Inf) \\
\mu_{v_e} &\sim& TN(2, 3, 0, Inf) \\
\mu_{sv_e} &\sim& TN(1, 1, 0, Inf) \\
\mu_A &\sim& TN(2, 2, 0, Inf) \\
\mu_b &\sim& TN(3, 3, 0, Inf) \\
\mu_{t_0} &\sim& TN(0.5, 0.5, 0, Inf) \\
\mu_{ELA} &\sim& TN(20, 20, 0, Inf) \\
\sigma_{v_c}, \sigma_{v_e} &\sim& \Gamma(1, 0.5) \\
\sigma_A, \sigma_b &\sim& \Gamma(1, 1) \\
\sigma_{t_0}, \sigma_{sv_e}, \sigma_{ELA} &\sim& \Gamma(0.2, 0.2) \\
\rho &\sim& U(-1, 0)\ \text{[First Model]} \\
\rho &\sim& TN(0, 0.1, -1, 0)\ \text{[Second Model]} \\
\rho &\sim& U(-1, 1)\ \text{[Estimation Model]}
\end{aligned}
$$

where ELA is the participants early life adversity score, BN is the bivariate normal, Γ is the gamma distribution, the first correlation distribution was used in the original joint model, and the second correlation distribution was used in the “tighter priors” model.

Distressing Life Events Items and Sources

Item	Source(s)
Acute illness (yourself) (i.e., any health condition with an abrupt onset and duration e.g., heart attack, pneumonia)	(AIHW, 2014; Australian Bureau of Statistics, 2014; Brim et al., 2000; Felitti et al., 1998)
Acute illness (household/family member) (i.e., any health condition with an abrupt onset and duration e.g., heart attack, pneumonia)	(AIHW, 2014; Australian Bureau of Statistics, 2014; Brim et al., 2000; Felitti et al., 1998)
Attempted suicide (household/family member)	(AIHW, 2014; Felitti et al., 1998)
Attempted suicide (self)	(AIHW, 2014; Felitti et al., 1998)
Chronic illness (yourself) i.e., any health condition lasting longer than 3 months requiring ongoing management e.g., diabetes, high blood pressure	(AIHW, 2014; Australian Bureau of Statistics, 2014; Brim et al., 2000; Felitti et al., 1998)
Chronic illness (family/household member) i.e., any health condition lasting longer than 3 months requiring ongoing management e.g., diabetes, high blood pressure	(AIHW, 2014; Australian Bureau of Statistics, 2014; Brim et al., 2000; Felitti et al., 1998)
Criminal Act (by self) (e.g., property damage/theft/graffiti)	
Criminal Act (by household/family member) (e.g., property damage/theft/graffiti)	(Australian Bureau of Statistics, 2014; Felitti et al., 1998)
Criminal Act (against self) (e.g., property damage/theft/graffiti)	(Australian Bureau of Statistics, 2014; Felitti et al., 1998)
Criminal Act (against household/family member) ((e.g., property damage/theft/graffiti)	(Australian Bureau of Statistics, 2014; Felitti et al., 1998)
Criminal Act (outside the home) (e.g., property damage/theft/graffiti)	(Australian Bureau of Statistics, 2014; Felitti et al., 1998)
Death (household/family member)	(Australian Bureau of Statistics, 2014)
Death (friend)	(Australian Bureau of Statistics, 2014)
Disability (yourself)	(Australian Bureau of Statistics, 2014; Brim et al., 2000)
Disability (household/family member)	(Australian Bureau of Statistics, 2014; Brim et al., 2000)
Divorce/Separation (Parent/Guardian)	(Australian Bureau of Statistics, 2014; Felitti et al., 1998)
Employment Termination/Retrenchment	(Australian Bureau of Statistics, 2014)
Gambling (yourself)	(Australian Bureau of Statistics, 2014)
Gambling (family/household member)	(Australian Bureau of Statistics, 2014)
Imprisonment/Institutionalisation (yourself)	
Imprisonment /Institutionalisation (family/household member)	(Felitti et al., 1998)
Mental illness (yourself)	(Australian Bureau of Statistics, 2014; Felitti et al., 1998)
Mental illness (household/family member)	(Australian Bureau of Statistics, 2014; Felitti et al., 1998)
Serious accident (yourself)	(Australian Bureau of Statistics, 2014; Felitti et al., 1998)
Serious accident (household/family member)	(Australian Bureau of Statistics, 2014; Felitti et al., 1998)
Serious accident (outside the home)	
Substance Abuse/Addiction (yourself)	
Substance Abuse/Addiction (family/household member)	(Australian Bureau of Statistics, 2014; Felitti et al., 1998)
Threatening/violent/abusive act(s) (against self)	(Australian Bureau of Statistics, 2014; Brim et al., 2000; Felitti et al., 1998)
Threatening/violent/abusive act(s) (against family/household member)	(Australian Bureau of Statistics, 2014; Brim et al., 2000; Felitti et al., 1998)
Threatening/violent/abusive act(s) (by family/household member)	(Australian Bureau of Statistics, 2014; Brim et al., 2000; Felitti et al., 1998)
Threatening/violent/abusive act(s) (outside the home)	
Verbal/emotional abuse (against yourself) (e.g., insulted, pestered, or intimidated)	(Australian Bureau of Statistics, 2014; Felitti et al., 1998)
Verbal/emotional abuse (against household/family member) (e.g., insulted, pestered, or intimidated)	(Australian Bureau of Statistics, 2014; Felitti et al., 1998)
Verbal/emotional abuse (outside the home) (e.g., people in the street being insulted, pestered, or intimidated)	
